# Application of extracellular vesicles proteins in cancer diagnosis

**DOI:** 10.3389/fcell.2022.1007360

**Published:** 2022-11-02

**Authors:** Defa Huang, Dingyu Rao, Xuxiang Xi, Zuxiong Zhang, Tianyu Zhong

**Affiliations:** ^1^ Laboratory Medicine, First Affiliated Hospital of Gannan Medical University, Ganzhou, China; ^2^ Department of Cardiothoracic Surgery, First Affiliated Hospital of Gannan Medical University, Ganzhou, China; ^3^ Precision Medicine Center, First Affiliated Hospital of Gannan Medical University, Ganzhou, China

**Keywords:** extracellular vesicles, proteins, cancer, diagnosis, biomarkers

## Abstract

Early tumor diagnosis is crucial for its treatment and reduction of death, with effective tumor biomarkers being important tools. Extracellular vesicles (EVs) are small vesicles secreted by cells with various biomolecules, including proteins, nucleic acids, and lipids. They harbor a double membrane structure. Previous studies on EVs in cancer diagnosis and therapy focused on miRNAs. Nonetheless, EVs contain proteins that represent physiological and pathological state of their parental cells. EVs proteins can reflect the pathological state of some diseases, which provides a basis for diagnosis and treatment. This study describes the role of EVs in cancer and summarizes the use of EVs proteins as diagnostic markers in different cancer types. Specifically, we discuss the potential and shortcomings of EVs as tumor biomarkers.

## Introduction

Cancer is the global leading cause of death. Early diagnosis is critical for its timely treatment and prognosis. Nevertheless, many cancers lack specific and effective diagnostic markers, resulting in missed treatment opportunities. Therefore, there is an urgent need for more effective and less invasive alternative markers for early diagnosis, individualized treatment strategies, and precise prognostic estimation.

Extracellular vesicles are a variety of membranous vesicles released by cells (Xiao et al., 2019). EVs secretion is mediated by hematopoietic and non-hematopoietic cells, including reticulocytes, B lymphocytes, T cells, epithelial cells, astrocytes, etc. (Laulagnier et al., 2004; Fader et al., 2005; Mignot et al., 2006). Besides, EVs have been detected in most body fluids, including urine, amniotic fluid, blood, serum, saliva, ascites, breast milk, cerebrospinal fluid, and nasal secretions (Keller et al., 2007; Li et al., 2008; Taylor and Gercel-Taylor, 2008). They regulate intercellular communication by transporting their contents. The EVs contents depict the phenotypic state of parental cells. EVs-mediated intercellular communication regulates normal physiological and pathological processes of several diseases, including cancer (Barteneva et al., 2013; Kucharzewska and Belting, 2013; Meckes, 2015).

EVs are found in most body fluids, highly stable with their contents similar to parental cells; therefore, they harbor significant potential as liquid biopsy specimens for various diseases (Rak, 2013; Hornick et al., 2015; Li et al., 2017a). Specifically, cancer-derived EVs may serve as biomarkers for early cancer detection since they carry biomolecules that indicate genetic or signaling alterations in the originating cancer cells (Li et al., 2015; Melo et al., 2015; Tang and Wong, 2015). Studies on the mechanisms by which EVs proteins regulate tumor progression and a summary of their feasibility as tumor markers have reached maturity. Nonetheless, studies on how to achieve the clinical use of EVs proteins in tumor diagnosis as well as prognostic assessment remain largely unexplored. Therefore, this review focuses on the use of EVs proteins in the diagnosis of various cancer types.

## Biogenesis and characterization of EVs

**FIGURE 1:**
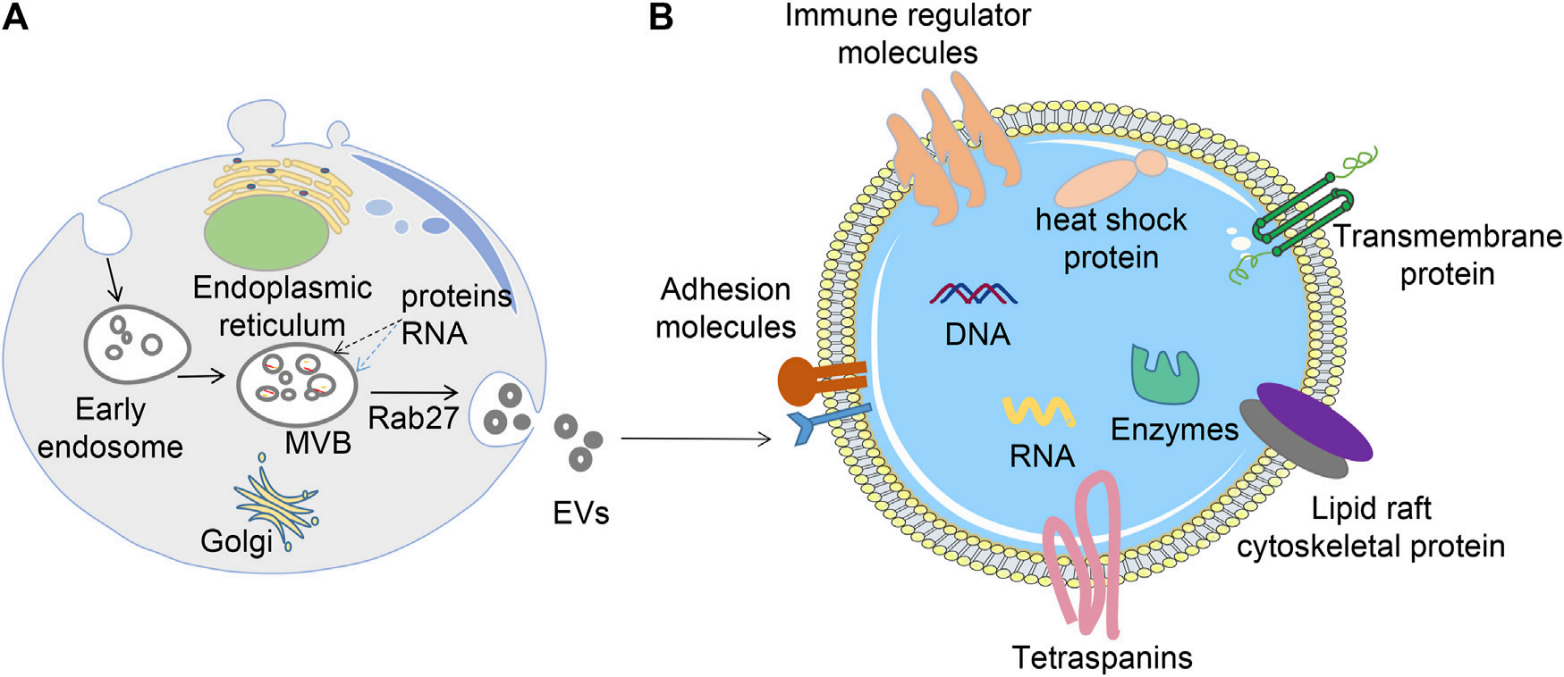
Biogenesis, release, and structure of EVs. **(A)** EVs are formed *via* the endosomal pathway and released upon fusion of MVBs with the plasma membrane; **(B)** EVs have a phospholipid bilayer membrane structure, the surface of the membrane contains many proteins, whereas the interior contains nucleic acids, proteins, and various enzymes.

Extracellular vesicles are a collective term for tiny vesicles with membrane structures that are actively secreted by cells. EVs were classified into exosomes, microvesicles and apoptotic vesicles depending on the formation process and size. Exosomes are formed by the fusion of multivesicular bodies with cell membranes and are 40–200 nm in diameter; microvesicles are formed by the outgrowth of cell membranes and are 200–2000 nm in diameter; apoptotic vesicles are formed by the atrophy and fragmentation of cells and are 500–2000 nm in diameter (Raposo and Stoorvogel, 2013). EVs are produced inside the cell through the endosomal pathway before being released into the extracellular space (Latifkar et al., 2019; Kalluri and LeBleu, 2020) (Figure 1A). First, the plasma membrane of the donor cell invaginates, forming early endonucleosomes. Thereafter, early endosomes mature into late endonucleosomes. During maturation, their membranes invaginate to form intraluminal vesicles (ILVs). Notably, endonucleosomes with ILVs are usually referred to as multivesicular bodies (MVBs). During the formation of MVB, bioactive molecules (e.g. proteins, mRNA, miRNA, lncRNA, and CircRNA) are packaged into the ILV by the endosomal sorting complex necessary for the transport (ESCRT)-dependent and ESCRT non-dependent pathways (Hessvik and Llorente, 2018). Eventually, ILVs are released into the extracellular space (EVs) when MVBs fuse with the plasma membrane. However, the mechanisms that drive EVs formation and secretion remain largely unknown due to different cell types and their states.

EVs carry various molecular contents hinged on their source and state (Duijvesz et al., 2011) (Figure 1B). For instance, EVs contain proteins associated with their biogenesis, including *CD9*, *CD63*, *CD81*, and *TSG101*, *Alix*, and *Rab* family proteins. All these molecules are routinely used as EVs marker proteins. Moreover, EVs harbor numerous nucleic acids, including mRNA, DNA, microRNA (miRNA), long non-coding RNA (LncRNA), etc. (Jeppesen et al., 2019). Also, lipids including cholesterol, phospholipids, glycerophospholipids, and sphingolipids are vital components of EVs. They form a bilayer structure and preserve their steady state (Skotland et al., 2017). At the same time, tumor cells may release more EVs into the microenvironment than normal cells, resulting in high levels of circulating EVs.

## The role of EVs in cancer

In cancer development, EVs-mediated intercellular communication is crucial in remodeling the tumor microenvironment and the formation of pre-metastatic ecological niches. Since tumor cells can establish strong communication with neighboring and distant cells, the tumor microenvironment (TME) modulates their growth and metastasis. The TME contains different components, including extracellular matrix (ECM), endothelial cells, cancer-associated fibroblasts (CAF), immune cells, and mesenchymal stem cells (Luga et al., 2012; Zhao et al., 2016; Mao et al., 2017; Morad and Moses, 2019; Thakur et al., 2020). Primary tumor cell-derived EVs induce the conversion of fibroblasts into metalloproteinase (MMP)-secreting myofibroblasts, in turn degrading ECM((Janowska-Wieczorek et al., 2005)). Additionally, these EVs stimulate neointima formation by activating macrophages in TME, hence generating an ecological niche for inflammation (Sanchez et al., 2016). Also, EVs induce epithelial-mesenchymal transition (EMT), during which epithelial cells lose their intercellular adhesion and separate from the tumor (Welton et al., 2010). This promotes the spread of cancer cells, i.e., one of the hallmarks of metastasis (An et al., 2015; Becker et al., 2016; Kim et al., 2020).

**FIGURE 2:**
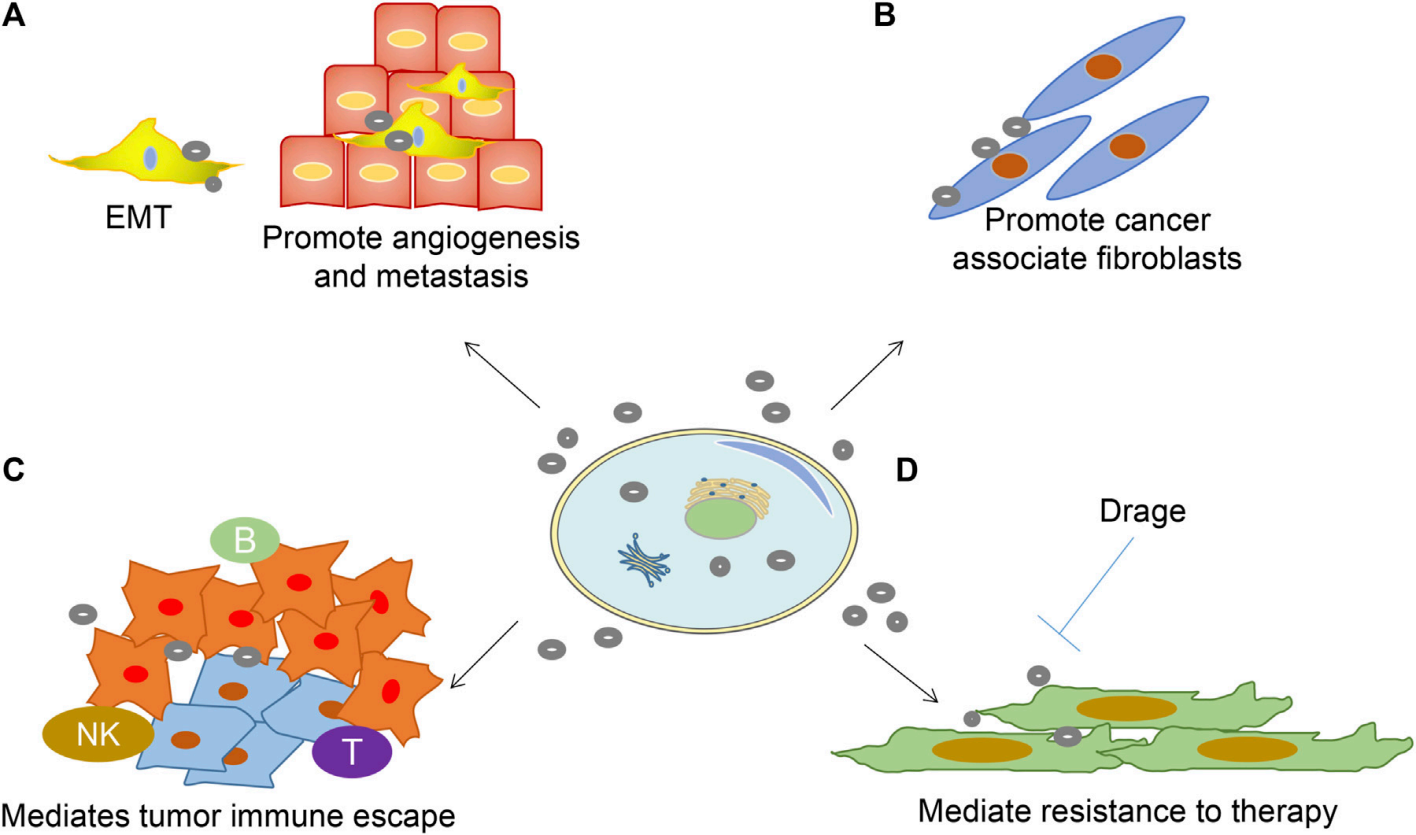
Role of EVs in sustaining cancer resistance networks. **(A)** EVs-released factors can promote EMT cell morphology, causing stemness and promoting angiogenesis;**(B)** Promote fibroblast-like cell formation that causes a desmoplastic reaction (stromal reaction); **(C)** Promote immune escape mechanisms; **(D)** EVs mediated resistance to treatment.

EVs regulate cancer progression, metastasis, and treatment outcomes. Besides, they promote cancer initiation, growth, progression, and resistance. EVs transfer oncogenic proteins and nucleic acids as well as interact with the tumor microenvironment (Zhang et al., 2015; Kosaka, 2016) (Figure 2). First, EVs promote angiogenesis and metastasis (Figure 2A). EVs uptake upregulates angiogenesis-related genes, resulting in enhanced endothelial cell proliferation, migration, and sprouting (Fraser et al., 2016). Cancer EVs are responsible for matrix activation, causing an angiogenic switch and increasing vascular permeability. Also, EVs promote metastasis by targeting epithelial-mesenchymal transition and forming a pre-metastatic ecological niche (Sceneay et al., 2013; Khalyfa et al., 2016). Secondly, EVs promote the formation of cancer-associated fibroblasts (Gu et al., 2012) (Figure 2B). With continuous supply, EVs from breast cancer cells MDA-MB 231 and glioblastoma cells U87 induce the transformation of recipient fibroblasts (Antonyak et al., 2011).

Furthermore, EVs mediate immune escape and production of an immunosuppressive environment, which is crucial in cancer pathogenesis (Figure 2C). EVs have been shown to induce apoptosis in cytotoxic T cells, regulatory T cell expansion, M2 polarization in macrophages, and suppression of cytotoxicity in natural killer cells (Condamine and Gabrilovich, 2011; Yang et al., 2012). Lastly, EVs shield cancer cells from the cytotoxic effects of chemotherapeutic agents and transfer chemoresistant properties to nearby cells (Wang et al., 2014) (Figure 2D). Stromal cell-derived EVs mediate the therapeutic resistance pathway in breast cancer cells by activating the pattern recognition receptor RIG-1 (Boelens et al., 2014).

## EVs proteins for diagnostic applications in cancer

EVs have attracted research interest due to their role in shuttling specific tumor markers in solid tumors. Unlike tumor-free individuals, cancer patients have higher concentrations of EVs proteins. Moreover, tumor EVs have robust information on cancer biology (Peinado et al., 2012). With the advent of proteomics techniques and means of EVs protein analysis, studies on EVs proteins have rapidly increased. Table 1 summarizes various body fluid-derived EVs proteins diagnosed in cancer.

**TABLE 1:** EVs secreted in different body fluids and their potential cancer markers.

Cancer type	Protein	Source	Sensitivity	Specificity	AUC	References
Lung cancer	CD9,CD63,CD81	Plasma	0.75	0.76	0.753	Jakobsen et al. (2015)
	LRG1	Urinary	-	-	-	Li et al. (2011)
	CD151	Plasma	-	-	0.68	Sandfeld-Paulsen et al. (2016)
	TSPAN8	Plasma	-	-	0.60	Sandfeld-Paulsen et al. (2016)
	Tim-3, Galectin-9	Plasma	-	-	-	Gao et al. (2018)
Nasopharyngeal carcinoma	LMP1, BARF1	Serum	-	-	-	Houali et al. (2007)
	Lmp1, LMP2A	Plasma	-	-	0.826	Hu et al. (2022)
Colorectal cancer	GPC1	Tissue,Plasma	-	-	-	Li et al. (2017b)
	CEA	Serum	0.875	0.975	0.884	Yokoyama et al. (2017)
Gastric cancer	ARG1,CD3,PD-L1,PD-L2	Plasma	-	-	-	Zhang et al. (2022)
Pancreatic cancer	GPC1	Serum	0.821	0.691	0.781	Melo et al. (2015)
	CA19-9	Serum	-	-	-	Sancho-Albero et al. (2020)
Cholangiocarcinoma	CD26, CD81, S1C3A1, CD10	Urinary	-	-	-	He and Zeng, (2016)
Prostate Cancer	PSA	Plasma	-	-	-	Logozzi et al. (2017)
	β-catenin, prostate cancer gene-3	Urinary	-	-	-	Nilsson et al. (2009)
Bladder cancer	TACSTD2	Urinary	-	-	-	Chen et al. (2012)
Ovarian cancer	CD24	Tissue	-	-	-	Runz et al. (2007)
	Claudin-4	Plasma	0.51	0.98	-	Li et al. (2009)
Spongioblastoma	EGFRV III	Serum	-	-	-	Skog et al. (2008)
Breast cancer	Survivin-2B	Serum	-	-	-	Khan et al. (2014)
Melanoma	CD63, Caveolin-1	Plasma	0.69	0.96	-	Logozzi et al. (2009)

### Respiratory cancers

#### Lung cancer

Lung cancer is one of the most prevalent human malignancies. At the time of diagnosis, nearly 70% of lung cancer patients present locally advanced or metastatic disease. Many studies have shown that EVs proteins are potential diagnostic markers for lung cancer. Jakobsen et al. developed an EV Array that coupled 37 antibodies targeting lung cancer-associated proteins and a panel of CD9, CD63, and CD81 antibodies to explore circulating EVs from healthy subjects and lung cancer patients. The authors used a combined 30-marker model EV Array, which can successfully distinguish the two groups with 75.3% accuracy. (Jakobsen et al., 2015). Li et al. identified human leucine-rich alpha-2-glycoprotein 1 (*LRG1*) in urinary EVs as a potential biomarker for NSCLC diagnosis. Based on proteomic mass spectrometry, *LRG1* accumulated in urinary EVs and was more highly expressed in NSCLC patients than in healthy individuals (Li et al., 2011). Elsewhere, Sandfeld et al. used 49 antibodies to detect EVs proteins obtained from 431 lung cancer patients and 150 healthy individuals (Sandfeld-Paulsen et al., 2016). Consequently, they noted that *CD151* and tetra-transmembrane protein 8 (TSPAN8) were more highly expressed in patients than in healthy individuals. Of note, *CD151* is also an independent biomarker in patients diagnosed with squamous cell carcinoma and small cell lung cancer. Gao et al. (Gao et al., 2018) showed that plasma EVs total protein, Tim-3, and Galectin-9 significantly increased in NSCLC, and are positively associated with larger tumor size, advanced TNM stage, and distant metastases. Therefore, EVs and their related components provide a theoretical foundation for research on molecular biomarkers for early lung cancer diagnosis.

### Nasopharyngeal carcinoma

Keryer et al. first detected latent membrane protein 1 (*LMP1*) in EVs of nasopharyngeal cell lines infected with Epstein Barr virus (EBV) (Keryer-Bibens et al., 2006). As a result, they discovered that nasopharyngeal carcinoma cells release EVs with galactose lectin 9 and *LMP1*, which inhibit T-cell viability. Houali et al. (Houali et al., 2007) analyzed LMP1 and Bam HI-A rightward frame 1(BARF1) proteins in the serum and saliva of young patients and adult nasopharyngeal carcinoma patients from North Africa and China. The results showed that both Both LMP1 and BARF1 were present in the serum and saliva from North African and Chinese patients with nasopharyngeal carcinomas (NPC). All young North African patients secreted both proteins, whereas 62% and 100% of adult patients secreted LMP1 and BARF1, respectively. They indicated that Both proteins will be a good diagnostic marker for NPC whereas BARF1 is a particularly promising marker for all ages of patients with NPC. For early diagnosis of nasopharyngeal carcinoma, Hu et al. recently combined EVs expressing the EBV-encoded membrane proteins LMP1 and LMP2A with EVs expressing other tumor marker proteins as liquid biopsy markers and significantly outperformed the traditional VCA-IgA assay in distinguishing patients with NPC from healthy donors and patients with nasopharyngitis, with accuracies of 96.3% and 83.1%. (Hu et al., 2022).

### Digestive system cancer

#### Colorectal cancer

Glypican-1^+^ (GPC1^+^) EVs were successfully isolated from tissues and plasma of Colorectal cancer (CRC). The percentage of GPC1^+^ EVs and the GPC1 protein expression in EVs from tumour tissues and plasma of CRC patients before surgical treatment was significantly elevated compared to that in the peritumoural tissues and the plasma of healthy controls. In conclusion, the increased plasma GPC1^+^ EVs expression is specific markers for the diagnosis of CRC (Li et al., 2017b). In contrast with serum carcinoembryonic antigen (CEA), serum EVs CEA predicts metastatic CRC with greater sensitivity and precision (Yokoyama et al., 2017). Silva et al. quantified plasma EVs in 91 patients diagnosed with colorectal cancer and found that EVs are significantly higher than that in controls; besides, these plasma EVs significantly correlate with CEA (56). As such, plasma EVs in patients with colorectal cancer act as tumor markers of disease progression and poor prognosis.

#### Gastric cancer

Gastric cancer (GC) is the fourth most common cancer and the second leading cause of cancer-related deaths across the globe (Silva et al., 2012). EVs from GC cells activate the *NF-ƙB* pathway in macrophages, thereby promoting cancer progression (Wu et al., 2016). Baran et al. discovered that the number of EVs is significantly higher in GC patients than that in normal controls (Baran et al., 2010). By exploring the expressional spectrum of plasma EV panel proteins in immune checkpoint inhibitor (ICI)‐treated GC, Zhang et al. identified EV‐derived ARG1/CD3/PD‐L1/PD‐L2 as biomarkers of ICI. Further, they combined them as an EV‐score that robustly predicts and dynamically monitors immunotherapeutic outcomes (Zhang et al., 2022).

#### Pancreatic cancer

Recent studies have shown that specific proteins are only detected in EVs derived from malignant cells. For instance, *GPC1*, a cell surface proteoglycan is overexpressed in breast and pancreatic cancers and only detected in EVs derived from these malignant cells. *GPC1*-positive EVs are diagnostic indicators of early pancreatic cancer (Melo et al., 2015). Circulating EVs with *GPC1* (*GPC1* ^*+*^ *Exos*) have been isolated from the blood of 250 pancreatic cancer patients, which distinguished patients with chronic pancreatitis from those with pancreatic cancer (early and advanced stages). In addition, *GPC1* ^*+*^ *Exos* can act as a preoperative and postoperative prognostic indicator. It is a significantly better prognostic marker for pancreatic cancer than *CA19-9*. Thus, *GPC1* ^*+*^ *Exos* can be utilized to diagnose early and advanced pancreatic cancer with high precision and sensitivity, as well as evaluate treatment. Albero et al. recently investigated the development of direct capture of *CA19-9* positive EVs from whole blood patients with high sensitivity for detecting *CA19-9* in EVs compared to serum samples (Sancho-Albero et al., 2020).

### Cholangiocarcinoma

Several oncogenic proteins are present in cholangiocarcinoma (CCA) human cell lines and the serum of patients with CCA, providing a basis for diagnosing cholangiocarcinoma. Additionally, *EGFR*, Mucin-1 (*MUC1*), and integrin beta-4 (*ITGB4*), which promote tumor growth and metastasis, are poor prognostic factors for this tumor (Arbelaiz et al., 2017). EVs concentration is also a useful biomarker in bile that discriminates malignant common bile duct (CBD) strictures from control or non-malignant CBD strictures with 100% accuracy (Severino et al., 2017). Furthermore, urinary EVs proteomics of mouse liver injury model identified 28 novel EVs closely related to disease, among which *CD26*, *CD81*, *S1C3A1,* and *CD10* are biomarkers of liver injury (He and Zeng, 2016).

### Genitourinary cancers

#### Prostate cancer

Plasma Prostate-specific antigen (PSA) is an extensively used biomarker for the detection and monitoring of prostate cancer (PCa). Nevertheless, PSA testing cannot distinguish between benign prostatic hypertrophy (BPH) and tumors (Hoffman, 2011). The acidity of the tumor microenvironment increases the EVs release and influences PSA in prostate cancer cells. PSA^+^ EVs in the plasma of PCa patients are four times higher than that of tumor-free controls (Logozzi et al., 2017). Additionally, γ-glutamyltransferase 1 (*GGT1*) is a cell surface enzyme s present in human serum EVs along with *CD9* (Kawakami et al., 2017). Nilsson et al. discovered that urinary EVs from prostate cancer patients express β-catenin, prostate cancer gene-3, a transmembrane serine protease, among other prostate cancer-related markers; this demonstrates the potential for diagnosis and monitoring of cancer patients (Nilsson et al., 2009).

#### Bladder cancer

Chen et al. conducted a comparative proteomic analysis of urinary EVs between nine hernia and nine bladder cancer (BCa) participants. Consequently, 107 proteins demonstrated differential expression between the two sample groups (Chen et al., 2012). In total, 24 proteins were significantly differentially expressed in 28 BCa and 12 hernia patients, with the area under the curve (AUC) of individual regions ranging between 0.702 and 0.896 and an AUC of 0.72 for tumor-associated calcium signal transducer 2 (TACSTD2). Elsewhere, Lee et al. performed proteomic identification of 1,222 proteins in urinary EVs between 10 healthy controls and 10 age-matched BCa patients; consequently, 56 proteins were significantly expressed in urinary EVs of BCa patients (Lee et al., 2018). This suggests that urinary EVs potentially provide an enrichment source for BCa protein biomarkers.

#### Ovarian cancer

Ovarian cancer is one of the fatal cancers, targeting women, with an estimated 70% of diagnoses happening at an advanced stage (Khalyfa et al., 2016). As such, the use of EVs contents for early diagnosis can potentially save many patients facing death due to late diagnosed ovarian cancer. The recent identification of epithelial cell adhesion molecules and *CD24* in ovarian cancer-derived EVs has emerged as a promising alternative for the early detection of ovarian cancer (Runz et al., 2007). Li et al. discovered that serum-derived EVs Claudin 4 progressively increased with cancer progression in ovarian cancer patients (Li et al., 2009). Szajnik et al. found that *L1CAM*, *CD24*, *ADAM10*, *EMMPRIN*, *TGFβ1*, *MAGE3/6,* and Claudin-4 in peripheral blood EVs can potentially be used for early diagnosis of ovarian cancer (Szajnik et al., 2013). EVs proteomics studies indicate that EVs in ovarian cancer are rich in integrin, *EGF* receptor, *Wnt* signaling, *PI3* kinase, *Fgf* receptor, *Ras*, *p53,* and angiogenic pathways among other proteins related to cancer genesis and development (Liang et al., 2013; Sinha et al., 2014). Comprehensive studies on the interactions between these molecules and their functions in signal transduction pathways may unravel the molecular mechanisms underlying malignant tumorigenesis and progression.

## Neurological diseases

### Spongioblastoma

Detecting serum EVs from 25 spongioblastoma patients reveals the presence of spongioblastoma-specific epidermal growth factor receptor variant type III (*EGFRV III*). As such, detecting EVs in cancer blood might provide diagnostic information and adjunctive therapy for cancer patients (Skog et al., 2008). Additionally, microfluidic microarrays are used to analyze the types of EVs proteins in the circulation of spongioblastoma patients. EVs with *EGFR-VII*, *EGFR*, *PDPN,* and *IDH1* secreted by spongioblastoma have been isolated, confirming that detection of circulating EVs predicts the clinical drug efficacy and cancer mutations (Shao et al., 2012).

## Other cancers

EVs proteins have been fronted as new diagnostic and prognostic indicators for various cancers. They may serve as biomarkers for breast cancer and melanoma. A significant increase in survivin levels has been reported in serum EVs from 40 breast cancer patients, and survivin-2B potentially acts as a diagnostic or prognostic marker for breast cancer (Khan et al., 2014). Melanoma-derived EVs promote metastasis by stimulating bone marrow-derived progenitor cells to prepare metastatic ecotopes. Wolfers et al. discovered that EVs secreted by melanoma harbor intact cancer antigens that activate CD8^+^ T cells and exhibit anticancer activity when absorbed by dendritic cells (Wolfers et al., 2001). Additionally, Logozzi et al. suggested that *CD63* and caveolin-1 in plasma EVs can act as a protein marker for melanoma (Logozzi et al., 2009).

## Discussion

Accumulating studies provide strong evidence for the use of these EVs-based protein markers for early cancer detection and even predict clinical outcomes. EVs proteins are directly derived from their secreting cells. EVs proteins obtained from cancer cells are emerging as novel biomarkers for cancer surveillance and efficacy evaluation according to the following characteristics (Xiao et al., 2019) Cancer-related lipids, proteins, RNA, and DNA in EVs can be used for cancer detection (Penfornis et al., 2016). (Mignot et al., 2006) EVs are small in size, can easily pass through the body tissue barrier, and are widely present in various body fluids, thereby easily detectable in clinical settings (Boukouris and Mathivanan, 2015). (Fader et al., 2005) The lipid bilayer membrane structure of EVs shields their contents from enzymatic degradation in blood circulation (Laulagnier et al., 2004). Blood composition is complex, and specific proteins secreted by cancer cells are diluted in the blood, therefore cancer proteins are not easily detectable at an early stage or low levels (He and Zeng, 2016). Of note, more than 10^9^ EVs are present in each milliliter of human blood. Based on these characteristics, the detection of EVs proteins has significant potential as a biomarker for cancer diagnosis and prognostic evaluation (Kugeratski et al., 2021). Rab GTPases, a large family of small GTPases that control membrane identity and EVs budding, uncoating, motility and fusion through the recruitment of effector proteins, such as sorting adaptors, tethering factors, kinases, phosphatases and motors (Stenmark, 2009). In addition, EVs proteins are used in cancer diagnosis as well as in a number of other diseases. Fraser et al. explored leucine-rich repeat kinase 2 (*LRRK2*) as a biomarker in urinary EVs obtained from patients with Parkinson’s disease and discovered that *ser-1292 LRRK2* is closely associated with PD (Fraser et al., 2016). Wang et al. (Wang et al., 2019) conducted a proteomic analysis of urine-derived EVs in PD patients vs. HC. Among all proteins discovered in urine- EVs, only two (*SNAP23* and calbindin), were highly expressed in PD patients vs. HC. Therefore, the expression of these two proteins potentially represents a valuable non-invasive biomarker for PD.

Nonetheless, obtaining pure and homogeneous EVs for comprehensive analysis remains a challenge, thereby limiting the clinical use of EVs proteins. The most difficult aspect of EVs research is their isolation and acquisition. At present, EVs are primarily obtained *via* ultracentrifugation (Coumans et al., 2017), precipitation (Mateescu et al., 2017), and immunocapture methods (Yamamoto et al., 2018); the former is unspecific enough for clinical use, whereas the latter may introduce bias and contamination of serum/plasma proteins. The development of reproducible isolation and extremely sensitive identification technologies effectively integrate data from various laboratories and improve their viability for clinical applications. ISEV recommends that each preparation of EVs be (Xiao et al., 2019) defined by quantitative measures of the source of EVs (e.g., number of secreting cells, volume of biofluid, mass of tissue); (Mignot et al., 2006) characterized to the extent possible to determine abundance of EVs (total particle number and/or protein or lipid content); (Fader et al., 2005) tested for presence of components associated with EV subtypes or EVs generically, depending on the specificity one wishes to achieve; (Laulagnier et al., 2004) tested for the presence of non-vesicular, co-isolated components (Thery et al., 2018). Excitingly, a method has been developed to capture EVs directly from plasma, serum or urine using a variety of EVs proteins. This method requires simple sample preparation without the need to isolate vesicles (Cho et al., 2019). Therefore, the future research strategies of EVs proteins may be divided into two types (Xiao et al., 2019) Isolation and purification of EVs for further study of EVs proteins. This method is limited by the difficulty of obtaining high purity sEVs with current technology (Mignot et al., 2006). Direct capture of EVs in body fluids by immunocapture method and use for protein analysis. However, this method requires antibodies specific for membrane proteins, and the sensitivity of the antibodies used and possible inhibitors of the reaction can affect the accuracy of the results. It is something to look forward to whether EVs in body fluids can be classified according to their proteins like blood cells. As such, the future use of EVs as early cancer detection and prognostic biomarkers of cancer will be a novel intervention to defeat cancer.
